# Digital lesson to convey the CanMEDS roles in general medicine using problem-based learning (PBL) and peer teaching

**DOI:** 10.3205/zma001357

**Published:** 2020-12-03

**Authors:** Andrea Winzer, Michael Jansky

**Affiliations:** 1 Universitätsmedizin der Johannes Gutenberg-Universität Mainz, Zentrum für Allgemeinmedizin und Geriatrie, Mainz, Germany

**Keywords:** general medicine, CanMEDS roles, interactive e learning units, online teaching, peer teaching, problem-based learning (PBL)

## Abstract

The CanMEDS roles has conveyed through a digital videoconference supported teaching format in which the students develop their own case-based, interactive e-learning units. The learning forms used are problem-based learning and peer-teaching. After a quality check by the lecturer the e-learning units are made available to further general medicine lessons.

## Zusammenfassung

Die Vermittlung der CanMEDS-Rollen erfolgt über ein digitales, Videokonferenz-gestütztes Lehrformat, in dem Studierende fallbasierte, interaktive Online-Lerneinheiten entwickeln. Als Lernformen kommen dabei problemorientiertes Lernen und Peer-Teaching zum Einsatz. Nach dozentenseitiger Qualitätsprüfung werden die digitalen Lerneinheiten weiteren allgemeinmedizinischen Unterrichten bereitgestellt.

## Introduction

Due to the limitation of classroom teaching caused by Covid-19 the Centre of General Medicine at the University of Medicine Mainz was faced with the challenge of teaching in virtual learning and teaching environments, staying in line with media-didactic concepts whilst at the same time remaining compliant with the licensing regulations in medical education. When switching to online teaching, not only should practice and patient oriented knowledge, abilities and, skills of general medicine be conveyed. Lesson concepts must also consider factors such as user friendliness, interactivity, motivation and use of multimedia. One particular challenge was the timely modification of the two elective compulsory modules “General Medicine Practice” and “Natural Medicine” in a one-week internship format. These modules mainly consist of practical exercises on patients in classroom teaching. In order to enable all students who were already enrolled to participate, both courses have been combined into an online elective compulsory module for students from the 3rd clinical semester onwards. With a problem-based teaching focus, knowledge of the CanMEDS roles should be transferred whilst the students develop their own interactive e-learning units, based on case vignettes of typical GP consultations.

## Project description

The superordinate learning objective of the new online elective module, with its pragmatism-based and creation-centred [1], [2] approach , is that students learn and apply the CanMEDS roles “Medical Expert”, “Communicator”, “Member of a Team” and “Scholar” [3], [http://www.nklm.de]. For this purpose, students should adopt the positions of reflective observers [4] as well as active practitioners in various teaching settings [5]. Subordinate specific learning objectives on a knowledge and comprehension level consist of acquiring knowledge about “general practices”, “practices of natural medicine” and “medical communication”. On the application and analysis level, students are required to develop a doctor-patient dialogue in a general practitioner context, taking into account therapy options of general and natural medicine. The design of interactive e-learning units takes place on the synthesis level [6].

In accordance with the university’s guidelines for pure distance learning, the videoconferencing method was chosen for learning tasks in plenary sessions and small groups [7], [8]. Problem-based learning (PBL) [9], [10], [11], cooperative and collaborative learning [12], [13], peer teaching [14], [15], [16] and independent learning by self-study [17], [18] were to be combined as didactic methods in order to achieve the intended increase in competence at the superordinate learning objective level [19], [20]. To determine whether the subordinate specific learning objectives had been achieved, the procedural task given to the students of designing interactive e-learning units [21], [22].

All interactive e-learning units should include a conclusive doctor-patient dialogue referring to a GP consultation. For this purpose, case vignettes with patient information (gender/age/occupation) and background information (aetiology, diagnostics, therapy) are provided. In order to design the dialogue, students must put themselves in the patient’s role (including the formulation of the patient's requests) as well as analyse the role of the GP (while bearing in mind a GP’s duties). By the end of the internship, each workgroup should have developed an interactive e-learning unit that can theoretically be used in further general medicine lessons.

The online elective compulsory module was evaluated using the standardized online questionnaire provided by the University of Medicine Mainz for the digital courses of the summer semester 2020, available in Moodle.

## Results

Table 1 (Tab. 1) shows the schedule for the case-based PBL-oriented teaching project, the application of the peer-teaching format and the CanMEDS role adoption by the students.

Upon completion of the elective compulsory module, the interactive e-learning units were reviewed with regard to their ability to be used further and, where necessary, revised by the course instructor based of his expertise as a professor for general medicine and as a general practitioner. All interactive e-learning units were still in use at the end of the summer semester within the digital hands-on training course in general medicine (8^th^ semester).

The new online teaching format was implemented without any problems in terms of organization and technology. According to the course administrator, the students' willingness to actively participate seemed to be more pronounced than in regular face-to-face teaching. In the concluding feedback session, the students reported a high level of comprehensibility with regard to the teaching concept and the learning objectives. The response rate of the online survey was 47% (9 of 19 participants). The course, the support, and the teaching format were rated as being very conducive to learning. The amount of subject matter was graded to be adequate.

The evaluation of the digital hands-on training course in general medicine had a response rate of 20.5% (36 of 175). In the free text comments the interactive e-learning units were rated as conducive to learning and requests for further such learning resources were expressed [23], [24].

## Discussion

Final conclusions cannot be drawn due to the small number of students attending the course and the response rate to the evaluation. Nevertheless, the feedback from students and the experiences of lecturers indicate that although the online teaching described cannot replace the process of acquiring competence by patient contact it can be a practical supplement. The digital teaching format is suitable to teach students the various CanMEDS roles with regard to family doctor activity, deepening available knowledge and expanding new competences at the application level. According to current data this is the result of its pragmatism-based and creation-centred approach as well as the opportunity related to acquire complex and sustainable competences via problem-based learning and peer-teaching [25], [26]. Thus, a learning transfer into practice can be achieved [27]. Furthermore, it allows the testing of innovative teaching-learning scenarios.

## Conclusion

The experiences identified with the online teaching format presented here will be incorporated into the development of our general medicine online teaching concept and encourage us to integrate online teaching formats more strongly in the future in the form of a teaching method mix. Thereby, the primary focus will remain on creating more room for teaching practical skills to patients in a face-to-face teaching environment. In addition, the level of transferability of the online teaching format presented should be part of an interdisciplinary review in the faculty, as well as in the exchange of experience with other chairs in general practice.

## Competing interests

The authors declare that they have no competing interests. 

## Figures and Tables

**Table 1:**
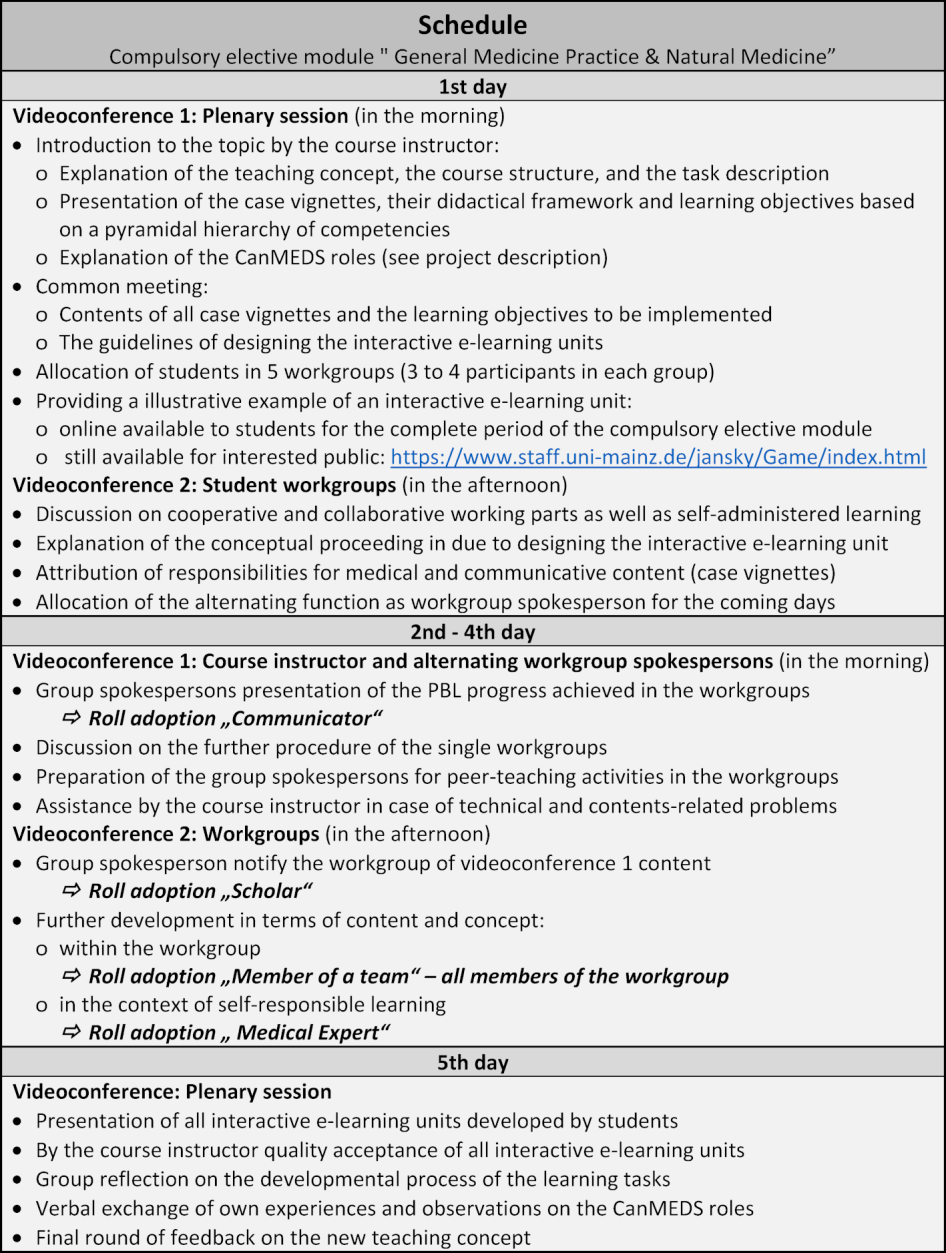
Schedule of the online compulsory elective module “General Medicine Practice & Natural Medicine” in the summer semester 2020 (compiled by A. Winzer)
